# Dengue Virus Infection Activates Interleukin-1β to Induce Tissue Injury and Vascular Leakage

**DOI:** 10.3389/fmicb.2019.02637

**Published:** 2019-11-22

**Authors:** Pan Pan, Qi Zhang, Weiyong Liu, Wenbiao Wang, Zhenyang Yu, Zizhao Lao, Wei Zhang, Miaomiao Shen, Pin Wan, Feng Xiao, Muhammad Adnan Shereen, Wen Zhang, Qiuping Tan, Yuntao Liu, Xiaohong Liu, Kailang Wu, Yingle Liu, Geng Li, Jianguo Wu

**Affiliations:** ^1^State Key Laboratory of Virology, College of Life Sciences, Wuhan University, Wuhan, China; ^2^Key Laboratory of Virology of Guangzhou, Institute of Medical Microbiology, Jinan University, Guangzhou, China; ^3^Center for Animal Experiment, Guangzhou University of Chinese Medicine, Guangzhou, China; ^4^Guangdong LongFan Biological Science and Technology, Foshan, China; ^5^Guangdong Provincial Hospital of Traditional Chinese Medicine, Guangzhou, China

**Keywords:** dengue virus, inflammatory response, IL-1RA, IL-1β, NLRP3 inflammasome, vascular leakage

## Abstract

Dengue virus (DENV) infection causes several diseases ranging from dengue fever to life-threatening dengue hemorrhagic fever and dengue shock syndrome characterized by endothelial dysfunction, vascular leakage, and shock. Here, we identify a potential mechanism by which DENV induces tissue injury and vascular leakage by promoting the activation of interleukin (IL)-1β. DENV facilitates IL-1β secretion in infected patients, mice, human peripheral blood mononuclear cells (PBMCs), mouse bone marrow-derived macrophages (BMDMs), and monocyte-differentiated macrophages (THP-1) *via* activating the NLRP3 inflammasome. The accumulated data suggest that IL-1β probably induces vascular leakage and tissue injury in interferon-alpha/beta receptor 1 deficient C57BL/6 mice (*IFNAR*^–/–^ C57BL/6), whereas IL-1 receptor antagonist (IL-1RA) alleviates these effects of IL-1β. Finally, administration of recombinant IL-1β protein results in vascular leakage and tissue injury in C57BL/6 mice. Together, the accumulated results demonstrate that IL-1β contributes to DENV-associated pathology and suggest that IL-1RA acts as a potential agent for the treatment of DENV-associated diseases.

## Introduction

The proinflammatory cytokine interleukin (IL)-1β is a regulator of systemic inflammatory response and functions as a fever-inducing pyrogen (Dinarello, 1997). IL-1β is activated by two distinct pathways. The nuclear factor (NF)-κB pathway facilitates the transcription of pro-IL-1β mRNA, whereas the second pathway involves the assembly of a multiprotein complex termed inflammasome that mediates pro-IL-1β maturation (Martinon et al., 2009). The inflammasome regulates the cleavage of inactive pro-IL-1β by activated caspase-1, a process that yields mature IL-1β (Kostura et al., 1989; Martinon et al., 2002; Allen et al., 2009; Rathinam and Fitzgerald, 2016). The best-characterized inflammasome, the pyrin domain-containing 3 (NLRP3), belongs to the Nod-like receptor family. As an essential part of the innate immune system, the inflammasome has a critical role in recognizing viral infection (Allen et al., 2009; Wang et al., 2018; Negash et al., 2019). Upon activation, NLRP3 recruits the apoptosis-associated speck-like protein, which contains a caspase recruitment domain (ASC), and procaspase-1 to form the inflammasome complex. Within the complex, caspase-1 is activated by autocatalytic cleavage to catalyze the conversion of pro-IL-1β into active IL-1β (Yan et al., 2015).

The particles of the dengue virus (DENV) are spherical, possess an envelope, and contain a single-stranded RNA genome of approximately 11 kb (Kuhn et al., 2002). The virus invades host cells by receptor-mediated endocytosis, and its RNA is translated into a polyprotein, which is then cleaved in three structural and seven non-structural proteins (Modis et al., 2004; Yu et al., 2008). Worldwide, DENV causes 390 million infections; 58.4 million of them are symptomatic, resulting in 13,586 deaths (Bhatt et al., 2013; Shepard et al., 2016). DENV infection causes several diseases, ranging from dengue fever to life-threatening dengue hemorrhagic fever and dengue shock syndrome (Guzman et al., 2010). Massive cytokine secretion (cytokine storm), heterotypic secondary infection, host genetics, and antibody-dependent enhancement (ADE) may contribute to DENV pathogenesis (Pang et al., 2007; Katzelnick et al., 2017). Despite the fact that dengue pathogenesis remains subtle, among them, the cytokine storm is believed to be one of the primary contributing factors (Hatch et al., 2011; Rothman, 2011). It has been demonstrated that DENV infection induces synthesis of IL-1β (Chang and Shaio, 1994; Hottz et al., 2013; Tan and Chu, 2013; Callaway et al., 2015), a cytokine typically generated at sites of injury or immunological challenge to coordinate cellular recruitment to the affected location (Puhlmann et al., 2005; Dinarello, 2010; Hakanpaa et al., 2018). However, the direct contribution of IL-1β to tissue injury in dengue disease remains elusive.

The present study documents that DENV induces tissue injury and vascular leakage by activating the NLRP3 inflammasome to release of IL-1β. In addition to previous findings, DENV was found to induce IL-1β activation in blood samples of infected patients, human peripheral blood mononuclear cells (PBMCs) and macrophages, C57BL/6 mice, and mouse bone marrow-derived macrophages (BMDMs). Moreover, DENV induces vascular leakage and tissue injury in mice most likely by activating IL-1β, whereas IL-1 receptor antagonist (IL-1RA) protects from this damage. Interestingly, recombinant IL-1β protein may directly induce vascular leakage and tissue injury in mice. Thus, the obtained results reveal the mechanism of DENV pathogenesis and provide evidence of a link between IL-1β and vascular leakage, thereby improving our understanding of the role of IL-1β in DENV pathogenesis.

## Materials and Methods

### Clinical Sample Analysis

All blood samples were provided by The First Affiliated Hospital of Guangzhou University of Chinese Medicine and Guangdong Province Traditional Chinese Medicine Hospital. Fifteen adult patients (eight males and seven females, median age 33 years) diagnosed with DENV infection were recruited in this study. The presence of DENV was confirmed by anti-dengue IgM and IgG enzyme-linked immunosorbent assay (ELISA) and qRT-PCR; the patients were negative for other pathogens. In addition, platelet count, hematological parameters (including hematocrit, hemoglobin, lymphocyte count, and monocyte count), and biochemical parameters (including aspartate aminotransferase, alanine aminotransferase, and alkaline phosphatase) were determined. Control samples were obtained from randomly selected 20 healthy individuals (10 males and 10 females, median age 35 years) with no history of DENV infection. Informed consent was obtained from each person (Supplementary Table 1). PBMCs were isolated by centrifugation in Histopaque (Haoyang Biotech) density gradient using fresh peripheral venous blood samples diluted 1:1 in pyrogen-free PBS. Separated PBMCs were washed twice with phosphate buffer saline (PBS) and resuspended in RPMI 1640 medium supplemented with 10% FBS, penicillin (100 U/ml), and streptomycin (100 μg/ml).

### Animal Studies

Wild-type (WT) C57BL/6 mice were purchased from the Hubei Research Center of Laboratory Animals (Wuhan, China). *IFNAR*^–/–^ C57BL/6 mice were provided by Prof. Jincun Zhao of Guangzhou Medical University, China. Both strains of mice were bred and maintained under specific pathogen-free conditions at Wuhan University. All protocols of animal experiments were reviewed and approved by the Institutional Animal Care and Use Committee of Wuhan University.

In all studies, age- and sex-matched mice were randomly assigned to experimental groups. For DENV2 infection assays, 6-week-old *IFNAR*^–/–^ C57BL/6 mice were tail vein injected with PBS (mock infection), pretreated with 300 μl PBS containing 2 μg mice IL-1RA by intraperitoneal injection for 90 min, treated with DENV2 (1 × 10^6^ PFU/mouse), and treated with IL-1RA (2 μg/mice) at 4 days postinfection again (DENV + IL-1RA infection) or infected with 300 μl containing 1 × 10^6^ PFU/mouse of DENV2 (DENV infection). One week after the DENV2 injection, mice were sacrificed, and tissues were collected for immunohistochemical and histopathological analyses.

BMDMs were isolated from 6–8-week-old male C57BL/6 mice. Cells were cultured for 6 days in RPMI 1640 medium supplemented with 10% fetal bovine serum (FBS) and 10% granulocyte-macrophage colony stimulating factor (GM-CSF)-conditioned medium collected from L929 cells.

### Ethics Statement

All human subjects used in this study were adults. The study was conducted according to the principles of the Declaration of Helsinki and approved by the institutional review board (IRB) of the College of Life Sciences, Wuhan University, in accordance with its guidelines for the protection of human subjects (IRB approval number AF/04-05.0/10.0). The collection of blood samples was conducted in accordance with the guidelines for the protection of human subjects. Written informed consent was obtained from each participant.

All animal studies were performed in accordance with the principles described by the Animal Welfare Act and the National Institutes of Health Guidelines for the care and use of laboratory animals in biomedical research. All experimental protocols involving mice were reviewed and approved by the Institutional Animal Care and Use Committee (IACUC) of the College of Life Sciences, Wuhan University (IACUC approval number WDSKY0201901). All of the mice that needed to be sacrificed used the method of euthanasia.

### Cell Lines and Cultures

Human monocytic cell line THP-1, *Aedes albopictus* gut cells (C6/36), and African green monkey kidney cells (Vero) were purchased from the American Type Culture Collection (ATCC). Human umbilical vein endothelial cells (HUVEC) were purchased from Obio Technology (Shanghai, China).

THP-1 cells were grown in RPMI 1640 medium supplemented with 10% FCS, 100 U/ml penicillin, and 100 μg/ml streptomycin sulfate. HUVEC, C6/36, and Vero cells were grown in dulbecco’s modified eagle medium (DMEM) with 10% FCS, 100 U/ml penicillin, and 100 μg/ml streptomycin sulfate. Cell cultures were kept at 37°C in a 5% CO_2_ incubator except for C6/36, which was maintained at 30°C.

THP-1 cells were stimulated with phorbol-12-myristate-13-acetate (PMA) for 12 h to induce differentiation into macrophages. The cells were stimulated with DENV, nigericin, lipopolysaccharide (LPS), and adenosine triphosphate (ATP). Supernatants were collected for the measurement of mature IL-1β (p17) and caspase-1(p20), while the cells were harvested for real time-polymerase chain reaction (RT-PCR) and immunoblot analyses.

### Viruses

The NGC strain of DENV2 (GenBank accession number KM204118.1) was kindly provided by Dr. Xulin Chen of Wuhan Institute of Virology, Chinese Academy of Sciences. The TSV01 strain of DENV2 (GenBank accession number AY037116.1) was kindly provided by Dr. Wenxin Li of College of Life Sciences, Wuhan University, China. To generate large stocks of the DENV, C6/36 cells or Vero cells were incubated with DENV2 at multiplicity of infection (MOI) of 0.5 for 2 h, and then the unbound DENV was washed away. Infected cells were cultured sequentially in a fresh medium with 2% FBS for 7 days. The supernatant was harvested and centrifuged at 4,000 rpm for 10 min to remove cellular debris and then filtered through a 0.22-μm membrane. The suspension of DENV was aliquoted and frozen at −70°C. The virus titer was determined by the plaque assay. For animal infection experiments, DENV was concentrated by centrifugation at 30,000 *g* for 2 h at 4°C and purified by centrifugation in a 20% sucrose solution at 80,000 *g* overnight at 4°C.

### Reagents and Antibodies

Phorbol-12-myristate-13-acetate (PMA), LPS, nigericin, ATP, and dansylsarcosine piperidinium salt (DSS) were purchased from Sigma-Aldrich. Caspase-1 inhibitor (VX-765) was purchased from Selleck. Recombinant human IL-1β protein (200-01B) and human IL-1RA (200-01RA) were obtained from Peprotech. Recombinant mouse IL-1β protein (401-ML/CF) and recombinant mouse IL-1RA (480-RM/CF) were purchased from R&D Systems. Trizol reagent was from Invitrogen, and Lipofectamine 2000 was from Invitrogen. The human IL-1β ELISA kit was purchased from BD Biosciences, the mouse IL-1β and tumor necrosis factor (TNF)-α ELISA kits were purchased from R&D Systems, and the LPS ELISA kit was purchased from Expandbio (Beijing, China).

Antibodies against NLRP3 (D4d8T), caspase-1 (D7F10), and IL-1β (D3U3E and 3A6) were purchased from Cell Signaling Technology. Antibody against DENV-NS3 (GTX124252) and DENV-Prm (GTX128092) were purchased from Genetex. Anti-β-actin antibody (66009) was purchased from Proteintech. Antibody against ASC (sc-271054) was purchased from Santa Cruz Biotechnology. Anti-mouse IgG Dylight 649, anti-mouse IgG Dylight 488, anti-rabbit IgG Dylight 649, and anti-rabbit IgG fluorescein isothiocyanate (FITC) were purchased from Abbkine.

### RNA Extraction and Quantitative RT-PCR

Total cellular RNA was extracted using the Trizol reagent (Invitrogen, Carlsbad, CA, United States) according to the manufacturer’s protocol. RNA (1 μg) was reverse-transcribed into cDNA by incubation with 0.5 μl of oligo(dT) and 0.5 μl of random primers, first at 37°C for 60 min and then at 72°C for 10 min. The resulting cDNA was used as the template for real-time PCR, performed in a LightCycler 480 thermal cycler (Roche). The thermocycling protocol included activation of polymerase at 95°C for 5 min, 45 cycles of 95°C for 15 s, 58°C for 15 s, and 72°C for 30 s; the fluorescence was measured and analyzed at the 72°C step. A final melting curve step from 50 to 95°C was applied to test the specificity of the primer. The primers used are listed in Supplementary Table 2.

### Enzyme-Linked Immunosorbent Assay (ELISA)

The concentration of culture supernatants and serum of IL-1β were measured by IL-1β ELISA Kit (BD Biosciences-CA, for human and R&D systems, Minneapolis, MN, United States, for mice), and the concentration of culture supernatants of LPS was measured by LPS ELISA Kit (EXPANDBIO) according to manufacturer’s instructions.

### Western Blotting

THP-1 cells induced to differentiate by PMA were washed twice with PBS and dissolved in lysis buffer (50 mM Tris–HCl, 150 mM NaCl, 0.1% Non-idet P-40, 5 mM EDTA, and 10% glycerol, pH 7.4). Protein concentration was measured using the Bradford assay kit (Bio-Rad, Richmond, CA, United States). The protein lysates, 50 μg, were electrophoresed in an 8–12% SDS-polyacrylamide gel and transferred to nitrocellulose membranes (Amersham, Piscataway, NJ, United States). Non-specific binding was blocked by incubating the membranes in 5% skim milk for 2 h. Subsequently, the membranes were then washed three times with PBS containing 0.1% Tween (PBST) and incubated with the antibody. Protein bands were visualized using a Luminescent Image Analyzer (Fujifilm LAS-4000).

### Immunofluorescence

THP-1 cells induced to differentiate by PMA were grown on sterile coverslips and infected with DENV2 (NGC) (MOI = 5) for 48 h. Subsequently, the cells were fixed with 4% paraformaldehyde for 15 min, washed three times with ice-cold wash buffer (PBS with 0.1% BSA), permeabilized with PBS containing 0.2% Triton X-100 for 5 min, and washed three times with the wash buffer. The cells were then blocked with 5% BSA for 30 min and incubated overnight with anti-ASC antibody and anti-DENV-Prm antibody (1:200 in wash buffer). Bound primary antibodies were visualized by staining for 1 h with FITC-conjugated donkey anti-mouse IgG and Daylight 649-conjugated donkey anti-rabbit IgG secondary antibody (1:100 in wash buffer). Nuclei were counterstained with DAPI for 5 min, and the cells were washed three times with the wash buffer. The immunostaining was examined by confocal microscopy (Fluo View FV1000; Olympus, Tokyo, Japan).

### ASC Oligomerization Analysis

THP-1 cells induced to differentiate by PMA were lysed in the lysis buffer. The lysates were gently agitated at 4°C for 30 min and centrifuged at 6,000 rpm at 4°C for 15 min. The pellets were washed three times with PBS and resuspended in 500 μl of PBS, and 2 mM disuccinimidyl suberate (DSS) (Sigma) was added. After cross-linking at 37°C for 30 min, the samples were centrifuged at 6,000 rpm for 10 min, and the cross-linked pellets were resuspended in 50 μl of 2 × SDS loading buffer. The suspension was boiled for 10 min and analyzed by Western blotting.

### Quantization of Vascular Leakage *in vivo*

One week after the infection with DENV2, the mice were injected intravenously with 300 μl of 0.5% Evans blue. The dye was allowed to circulate for 2 h, and the mice were euthanized and extensively perfused with PBS. Tissues were collected, weighed, and incubated in 1 ml formamide and 37°C for 24 h. The concentration of Evans blue concentration was measured as OD_610_ and calculated from the standard curve. Data were expressed as (ng Evans blue)/(mg tissue weight).

### *Trans-*Endothelial Electrical Resistance

Human umbilical vein endothelial cells monolayers were grown on a Transwell polycarbonate membrane system (Corning Inc.) and treated with recombinant human IL-1β or preincubated with IL-1RA for 24 h. Half of the medium contained in the upper and lower chambers was replaced by fresh endothelial cell medium. Untreated HUVECs were used as a negative control, and medium alone was used as a blank control. Endothelial permeability was evaluated by measuring *trans-*endothelial electrical resistance (TEER) at 2-h intervals using the EVOM2 epithelial Voltohmmeter (World Precision Instruments). Relative TEER was expressed as follows: [(resistance in experimental group) – (resistance in medium alone)]/[(resistance in untreated HUVECs) – (resistance in medium alone)]; resistance was expressed in ohms.

### Statistical Analysis

All experiments were repeated at least three times with similar results. All results were expressed as the mean ± the standard error of mean (SEM). Statistical significance of the difference between two groups was calculated using the *t*-test and among multiple groups by one-way ANOVA. GraphPad Prism5 software was used to perform statistical analyses. *P*-values of less than 0.05 were considered statistically significant.

## Results

### DENV Infection Activates IL-1β in Humans and Mice

**FIGURE 1:**
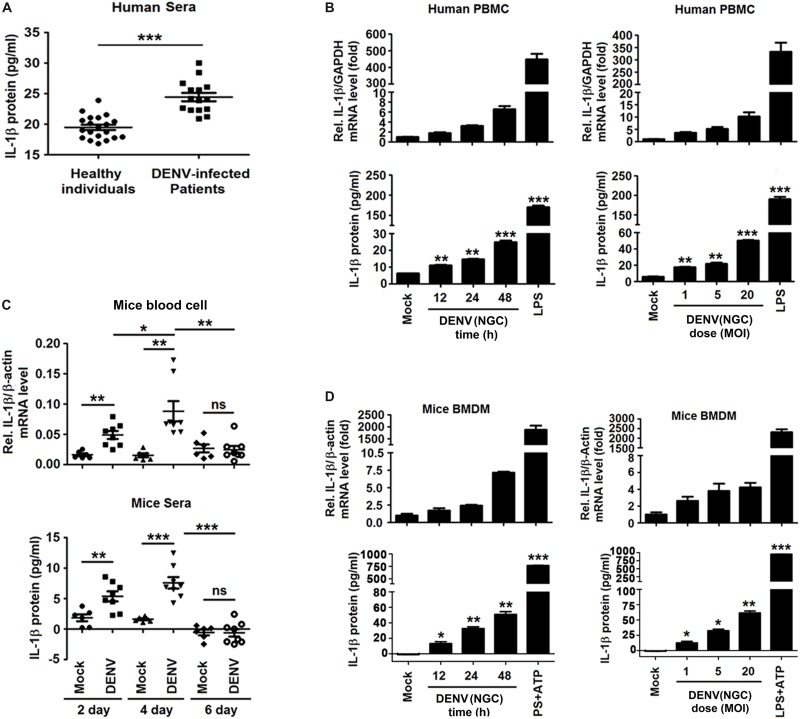
Dengue virus (DENV) infection activated interleukin (IL)-1β in patients and mice. **(A)** Blood samples were obtained from 15 DENV-infected patients and 20 healthy individuals. The serum level of IL-1β was measured by ELISA. Points represent the IL-1β value in each serum sample. **(B)** Human PBMCs were infected with DENV2(NGC) for 12, 24, and 48 h (left), different concentrations of the virus (MOI = 1, 5, and 20) for 24 h (right), or treated with LPS (1 μg/ml) for 6 h. The intracellular level of IL-1β mRNA was determined by qRT-PCR analysis (top), and IL-1β protein in cell culture supernatant was measured by ELISA (bottom). **(C)** *IFNAR*^–/–^ mice were infected with DENV2(NGC) (*n* = 8) or mock-infected (*n* = 6), and blood samples were collected at 2, 4, and 6 days later. IL-1β mRNA in blood cells was determined by qRT-PCR (top), and the serum level of IL-1β protein was measured by ELISA (bottom). Points represent the individual IL-1β value in each serum sample. **(D)** Mouse BMDMs differentiated by GM-GSF were infected with DENV2(NGC) for 12, 24, and 48 h at MOI = 5 (left) or at different MOI (MOI = 1, 5, and 20) for 24 h (right), treated with LPS (1 μg/ml) for 6 h, or stimulated with ATP (10 mM) for 20 min. IL-1β mRNA was determined by qRT-PCR (top), and IL-1β protein was measured by ELISA (bottom). Mock: PBS or supernatant of C6/36 cells without DENV2 infection. The number of replicates equals 2 **(A,C)** or 3 **(B,D)**. Values are mean ± SEM; ns, not significant; ^∗^, ^∗∗^, ^∗∗∗^ indicate *P*-values less than 0.05, 0.01, and 0.001, respectively.

To determine whether DENV infection affects the level of circulating IL-1β, blood samples of DENV-infected patients (*n* = 15) and healthy individuals (*n* = 20) were analyzed (Supplementary Tables 1,3). Serum level of IL-1β was significantly higher in dengue patients than in healthy individuals (24.44 ± 0.68 pg/ml vs. 19.48 ± 0.42 pg/ml, *P* < 0.0001) (Figure 1A). Infection of human PBMCs by DENV2(NGC) (Liu et al., 2014; Zhu et al., 2017) increased the expression of IL-1β at the mRNA and protein levels (Figure 1B). DENV2(NGC) *E* mRNA and infectious DENV2 (NGC) were confirmed by qRT-PCR and plaque assays, suggesting that DENV successfully infected PBMCs (Supplementary Figures 1A,B). The effect of DENV2(NGC) infection on the secretion of IL-1β was then evaluated in *IFNAR*^–/–^ C57BL/6 mice deficient in IFN-α/β receptor (Beatty et al., 2015). IL-1β mRNA in blood cells and IL-1β protein in the sera were higher in DENV2(NGC)-infected mice (*n* = 8) than in mock-infected mice (*n* = 6) (Figure 1C). The presence of DENV2(NGC) *E* mRNA in the blood cells of mice was verified, suggesting that DENV successfully infected DENV2(NGC)-infected mice (Supplementary Figure 1C). The level of IL-1β mRNA and IL-1β protein in BMDMs of C57BL/6 mice was increased after DENV2(NGC) infection (Figure 1D). Infected BMDMs produced DENV2(NGC) *E* mRNA and infectious DENV(NGC), suggesting that DENV successfully infected BMDMs (Supplementary Figures 1D,E). In all above-described experiments, LPS or LPS + ATP-stimulated cells (Figures 1B,D) were used as positive controls. Collectively, these results demonstrate that DENV induces IL-1β activation in infected patients and human PBMCs, as well as in *IFNAR*^–/–^ C57BL/6 mice and BMDMs isolated from these animals. These findings expand the conclusions of previous reports (Hottz et al., 2013; Wu et al., 2013).

### Different Strains of DENV Activate IL-1β in THP-1 Macrophages

**FIGURE 2:**
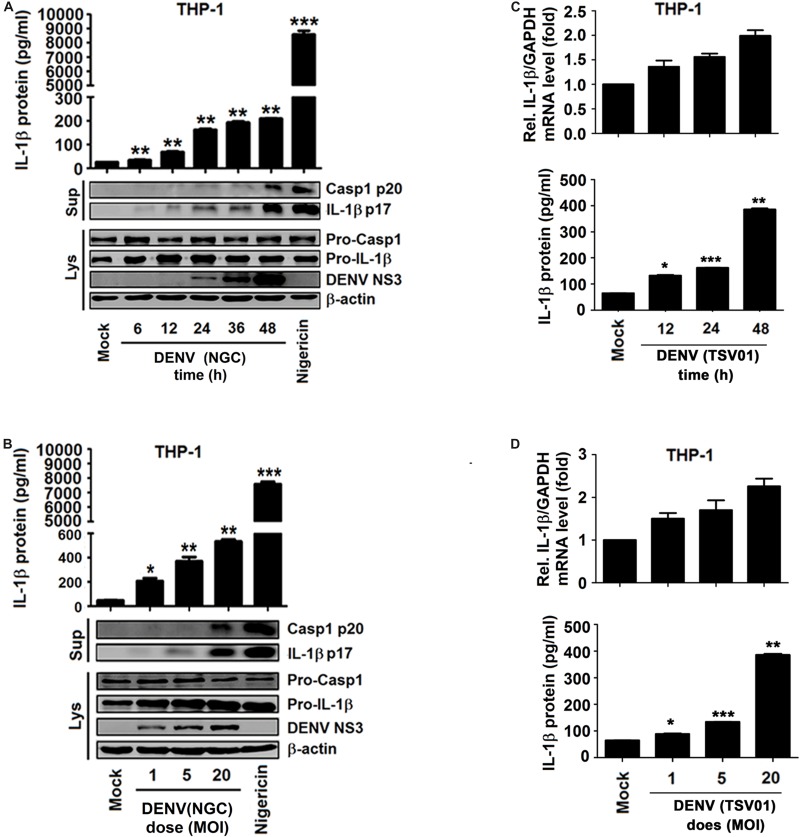
Different strains of dengue virus (DENV) activate interleukin (IL)-1β in THP-1 macrophages. **(A,B)** Macrophages differentiated from THP-1 cells by phorbol-12-myristate-13-acetate (PMA) were infected with DENV2(NGC) for 6, 12, 24, 36, and 48 h at MOI = 5 **(A)** or at different MOI (MOI = 1, 5, and 20) for 24 h **(B)** or treated with 2 μM nigericin for 90 min. The secreted IL-1β was measured in cell supernatant by ELISA (top), and intracellular proteins (LYS) were analyzed by Western blotting (WB) (bottom). **(C,D)** Macrophages differentiated from THP-1 cells by PMA were infected with DENV2 (TSV01) for 12, 24, and 48 h at MOI = 5 **(C)** or at different MOI (1, 5, and 20) for 24 h **(D)**. IL-1β mRNA was determined by qRT-PCR (top), and IL-1β protein was measured by ELISA (bottom). Mock: supernatant of C6/36 cells without DENV2 infection. *N* = 3 in all experiments. Values are mean ± SEM. ^∗^, ^∗∗^, ^∗∗∗^ indicate *P*-values less than 0.05, 0.01, and 0.001, respectively.

**FIGURE 3:**
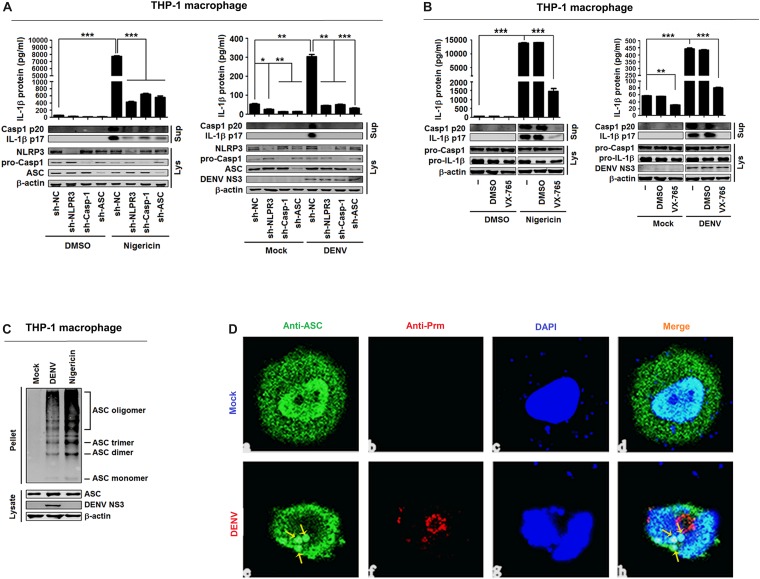
Dengue virus (DENV) infection induces interleukin (IL)-1β release by activating the NLRP3 inflammasome. **(A)** Macrophages differentiated from THP-1 cells by phorbol-12-myristate-13-acetate (PMA) stably expressing sh-RNAs, sh-*NLRP3*, sh-*ASC*, sh-*Casp-1*, or sh-NC were stimulated by 2 μM nigericin for 2 h (left) or infected with DENV2 at MOI = 5 for 24 h (right). IL-1β protein in cell supernatant was measured by ELISA (top), and intracellular proteins (LYS) were analyzed by Western blotting (bottom). **(B)** Macrophages differentiated from THP-1 cells by PMA were stimulated by 2 μM nigericin for 2 h. IL-1β protein in cell supernatant was measured by ELISA (top), and intracellular proteins (LYS) were analyzed by Western blotting (bottom). **(C)** Macrophages differentiated from THP-1 cells by PMA were stimulated by 2 μM nigericin for 2 h (left) or infected with DENV2 at MOI = 5 for 24 h (right). ASC oligomerization in cytosolic pellets cross-linked with disuccinimidyl suberate (DSS) was analyzed by immunoblotting. **(D)** Macrophages differentiated from THP-1 cells by PMA were infected with DENV2 at MOI = 5 for 24 h. The subcellular localization of ASC (green) and DENV2 (red) was visualized with confocal microscopy. sh-NC: negative control vector containing scrambled shRNA. Mock: macrophages treated with the supernatant of C6/36 cells without DENV2 infection. *N* = 3 in all experiments. Values are mean ± SEM. ^∗^, ^∗∗^, ^∗∗∗^ indicate *P*-values less than 0.05, 0.01, and 0.001, respectively.

Whether different strains of DENV2 affect the synthesis and secretion of IL-1β was evaluated in macrophages generated by PMA-induced differentiation of THP-1 cells (Park et al., 2007). Infection by DENV2(NGC) resulted in the activation of IL-1β secretion, Casp-1 p20 maturation, IL-1β p17 processing, and DENV2 NS3 production (Figures 2A,B). The increase in the level of pro-IL-1β in cell lysates raised the possibility that DENV infection could activate the NF-κB signaling pathway, upregulating the expression of IL-1β mRNA. This mechanism is supported by data shown in Figures 2C,D. The level of procaspase-1 in cell lysates remained essentially unchanged, and the significant increase in IL-1β and caspase-1 in the supernatant resulted from the activation of NLRP3 inflammasome by DENV infection (Figure 3). The NLRP3 inflammasome can cleave procaspase-1 to generate active caspase-1, which, in turn, can convert pro-IL-1β into active IL-1β. DENV2 (NGC) *E* mRNA and the infectious virus were detected in infected cells (Supplementary Figures 2A,B). IL-1β mRNA and IL-1β protein were also induced upon infection by the TSV01 strain of DENV2 (Grant et al., 2011; Figures 2C,D). DENV2 (TSV01) *E* mRNA and infectious virus were detected in infected cells (Supplementary Figures 2C,D). Importantly, LPS (Chen and Wang, 2002) was not detected in mock solution and stocks of DENV2(NGC) and DENV2(TSV01) (Supplementary Figure 2E), excluding the possibility that the observed effects could be caused by LPS contamination. Together, the results demonstrated that both DENV2(NGC) and DENV2(TSV01) activate synthesis, maturation, and secretion of IL-1β.

### DENV Induces IL-1β Release *via* Activating the NLRP3 Inflammasome

IL-1β activation is regulated at two levels: the transcription of pro-IL-1β mRNA and the processing of IL-1β protein (Martinon et al., 2002), and the maturation and release of IL-1β are controlled by inflammasomes (Swanson et al., 2019). Therefore, experiments were performed to determine whether the NLRP3 inflammasome is involved in DENV-induced activation of IL-1β. For this purpose, THP-1 cells stably expressing short hairpin RNAs (shRNAs) attenuating the mRNAs of NLRP3 inflammasome components NLRP3, Casp-1, and ASC were generated (Wang et al., 2017) (Supplementary Figures 3A–C). The cells were then differentiated into macrophages and stimulated with nigericin, an inducer of Casp-1 maturation and IL-1β release, or infected with DENV2(NGC) (Ito et al., 2012). Both types of stimulation increased IL-1β secretion, IL-1β p17 processing, and Casp-1 p20 maturation, but these effects were attenuated by stable expression of sh-NLRP3, sh-Casp-1, and sh-ASC (Figure 3A). These findings indicate that knockdown of NLRP3 inflammasome components attenuates nigericin- and DENV2-induced IL-1β activation and caspase-1 maturation. Next, macrophages were derived from THP-1 cells with VX-765, a specific inhibitor of caspase-1 (Stack et al., 2005), and stimulated with nigericin or infected with DENV2(NGC). Nigericin- and DENV2(NGC)-induced IL-1β secretion, IL-1β (p17) processing, and caspase-1 (p20) maturation were all inhibited by VX-765 (Figure 3B), indicating that caspase-1 has a critical function in the DENV-induced processing of IL-1β. NLRP3 recruits ASC to participate in the maturation of caspase-1, and ASC oligomerization indicates the activation of inflammasome (Swanson et al., 2019). As illustrated in Figure 3C, nigericin and DENV2(NGC) induced oligomerization of ASC. ASC was diffusely distributed in mock-infected macrophages but formed small specks, indicative of oligomerization, in DENV-infected macrophages (Figure 3D). Therefore, the possibility can be raised that DENV2 activates NLRP3 inflammasome to induce IL-1β processing. These findings are consistent with an earlier report (Hottz et al., 2013).

### DENV Promotes IL-1β Release in *IFNAR*^–/–^ C57BL/6 Mice

**FIGURE 4:**
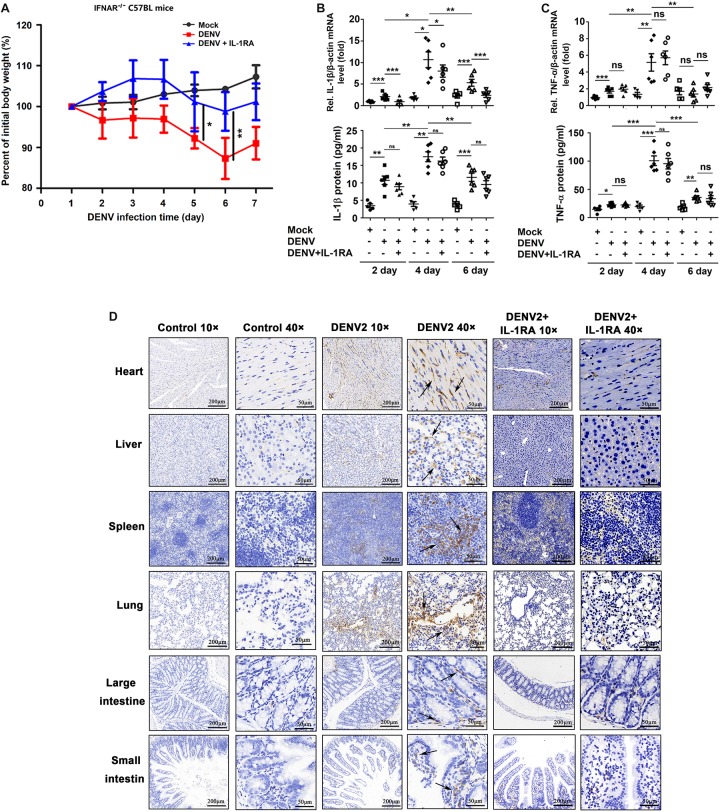
Dengue virus (DENV) promotes interleukin (IL)-1β release in *IFNAR*^–/–^ C57BL/6 mice. **(A–D)** *IFNAR*^–/–^ C57BL/6 mice were intravenously injected with 300 μl of DENV2(NGC) suspension at a dose of 1 × 10^6^ PFU/mouse (*n* = 6), pretreated with intraperitoneal injection of 300 μl of PBS containing 2 μg of mouse IL-1RA at 90 min before the infection with DENV2(NGC) (1 × 10^6^ PFU/mouse) with the IL-1RA treatment repeated 4 days after the infection (*n* = 6) or injected with 300 μl of PBS only (control group, *n* = 6). Mice were euthanized 7 days after infection or PBS injection, and the tissues were collected. **(A)** Mice were weighed daily; body weight is expressed as the percentage of the initial weight. **(B)** Blood samples were collected at 2, 4, and 6 days postinfection. IL-1β mRNA in blood cells was determined by qRT-PCR (top), and IL-1β protein in the serum was measured by ELISA (bottom). Individual points represent the IL-1β value in each sample. **(C)** Blood samples were collected at 2, 4, and 6 days postinfection. TNF-α mRNA in blood cells was determined by qRT-PCR (top), and TNF-α protein in the serum was measured by ELISA (bottom). Individual points represent the TNF-α value in each sample. **(D)** Detection of IL-1β by immunohistochemistry in the heart, liver, spleen, lung, large intestine, and small intestine after DENV infection. Black arrows indicated the immunostaining of IL-1β. Mock: injection of the same volume of PBS. Data represent two independent experiments. Values are mean ± SEM; ns, not significant; ^∗^, ^∗∗^, ^∗∗∗^ indicate *P*-values less than 0.05, 0.01, and 0.001, respectively.

To determine whether a cause-and-effect relationship exists between the activation of IL-1β and vascular leakage *in vivo*, an *IFNAR*^–/–^ C57BL/6 mouse model was generated, and the animals were infected with DENV(NGC). These experiments were performed since previous studies reported vascular leakage in AG129 or *STAT1*^–/–^ mice infected with the DENV2(NGC) strain (Johnson and Roehrig, 1999; Chen et al., 2008; Sung et al., 2019) and in *IFNAR*^–/–^ mice infected with the DENV2 (PL046) strain (Beatty et al., 2015), but the effects of infection of *IFNAR*^–/–^ C57BL/6 mice with the DEV2(NGC) strain were not studied. In the present experiments, *IFNAR*^–/–^ C57BL/6 mice were treated with PBS (*n* = 6), infected with DENV2(NGC) (*n* = 6), or injected intravenously with IL-1RA (Collison, 2019) and then infected with DENV2(NGC) (*n* = 6). While the body weight of mock-infected mice gradually increased, DENV2(NGC)-infected mice lost weight from 1 to 6 days postinfection, and the body weight of DENV2(NGC)-infected mice treated with IL-1RA increased from 1 to 3 days postinfection and gradually decreased during the infection (Figure 4A). The body weight of DENV2-infected mice treated with IL-1RA was significantly higher than in the absence of IL-1RA treatment (Figure 4A), suggesting that IL-1RA may compensate for the loss of body weight induced by DENV2. High levels of DENV2 *E* mRNA (Supplementary Figures 4A,B), NS5 mRNA (Supplementary Figures 4C,D), and viral copies (Supplementary Figures 4E,F) were detected in DENV2-infected mice serum and tissues, including heart, liver, spleen, lung, kidney, large intestine, and small intestine, but were not identified in mock-infected mice. These data indicate that the virus successfully infected the host organism. Interestingly, the levels of DENV *E* mRNA, NS5 mRNA, and viral copies were lower in DENV2-infected mice treated with IL-1RA than in untreated infected mice (Supplementary Figures 4A–F), suggesting that IL-1RA may inhibit DENV2 infection. At 4 days after the infection with DENV, blood level of IL-1β and TNF-α mRNA and protein was increased (Figures 4B,C) in DENV2-infected mice and DENV2-infected mice treated with IL-1RA but not in mock-infected mice. Moreover, IL-1β was upregulated in the heart, liver, spleen, lung, large intestine, and small intestine of DENV2-infected mice but not in the organs of mock-infected mice or DENV2-infected mice treated with IL-1RA (Figure 4D). Together, these results demonstrate that DENV activates the synthesis and secretion of IL-1β and TNF-α in *IFNAR*^–/–^ C57BL/6 mice.

### DENV Induces Tissue Injury and Vascular Leakage in Mice

**FIGURE 5:**
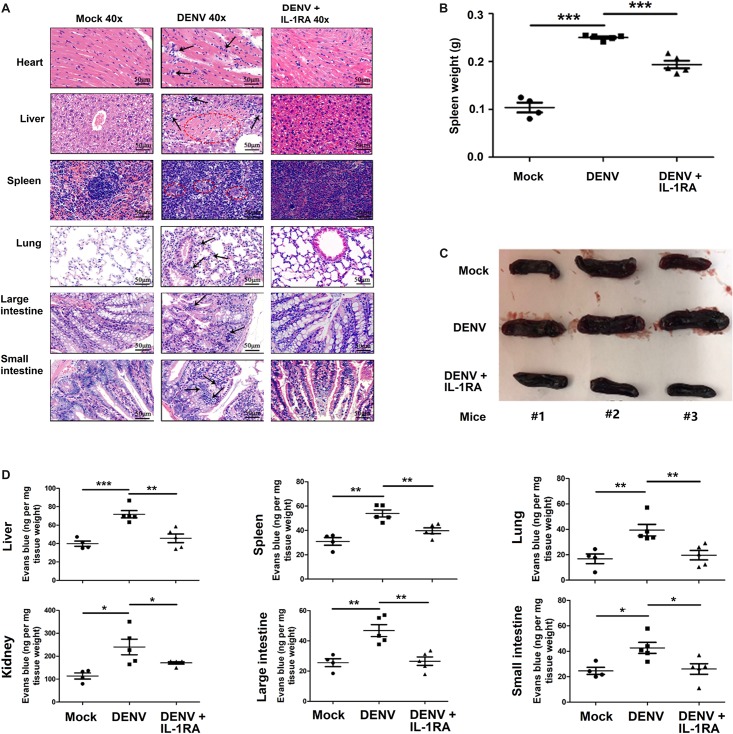
Dengue virus (DENV) induces tissue injury and vascular leakage in mice. **(A–D)** *IFNAR*^–/–^ C57BL/6 mice were intravenously injected with 300 μl of DENV2 suspension at a dose of 1 × 10^6^ PFU/mouse (*n* = 6), pretreated with intraperitoneal injection of 300 μl of PBS containing 2 μg interleukin (IL)-1RA at 90 min before injection of DENV2(NGC) (1 × 10^6^ PFU/mouse); the IL-1RA treatment was repeated 4 days after the viral infection (*n* = 6) or injected with 300 μl of PBS (control group, *n* = 6). Mice were euthanized 7 days after infection, and histopathologic analysis of the heart, liver, spleen, lung, large intestine, and small intestine was performed. Black arrows indicate the infiltrated inflammatory cells; the red circle indicates the aberrant cells **(A)**. Weight changes **(B)** and anatomy **(C)** of the spleen in the three groups of mice. Mice were intravenously injected with Evans blue 7 days after infection. DENV-infected group, *n* = 5; DENV + IL-1RA group, *n* = 5; control group, *n* = 4. Mice were euthanized after the dye was circulating for 2 h, and the concentration of Evans blue was measured at OD_610_ in the liver, spleen, lung, kidney, large intestine, and small intestine **(D)**. Data represent two independent experiments. Values are mean ± SEM; ^∗^, ^∗∗^, ^∗∗∗^ indicate *P*-values less than 0.05, 0.01, and 0.001, respectively.

In agreement with the above results, inflammatory cell infiltration and tissue injury were present in organs of DENV2-infected C57BL/6 mice but not in organs of mock-infected mice (Figure 5A). Notably, in the spleen of DENV2-infected mice, but not in the spleen of infected mice treated with IL-1RA, the boundaries between the red and the white pulp were disrupted, and the number of lymphatic nodules and pulping cells was increased, resulting in a significant infiltration of lymphocytes into the red pulp region (Figure 5A). The spleen weight and size in infected mice were significantly higher than in mock-infected mice or infected mice treated with IL-1RA (Figures 5B,C). Given the major function of the spleen in the immune system (Mebius and Kraal, 2005), DENV2-induced surge of inflammatory cytokines might have generated considerable damage to the spleen. Thus, the results appear to indicate that DENV2 induces IL-1β secretion and inflammatory responses in mice and may contribute to inflammatory cell infiltration and tissue injury after DENV infection in mice. Additionally, the intensity of Evans blue dye in the liver, spleen, lung, kidney, large intestine, and small intestine of DENV2-infected mice was significantly higher than in mock-infected mice or DENV2-infected mice treated with IL-1RA (Figure 5D). These results probably suggest that DENV2 infection in mice induces inflammatory response and vascular leakage by an IL-1β-dependent mechanism.

### Recombinant IL-1β Protein Induces Vascular Leakage in Mice

**FIGURE 6:**
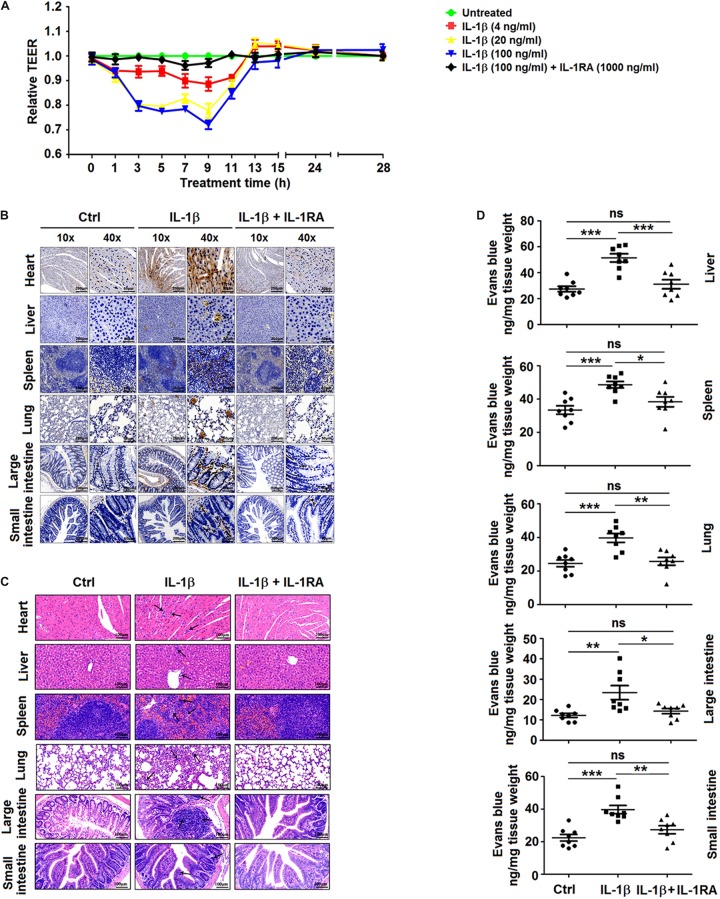
Recombinant interleukin (IL)-1β induces vascular leakage in mice. **(A)** Confluent monolayers of human umbilical vein endothelial cells (HUVEC) were grown on polycarbonate membrane system and treated with different concentrations of recombinant human IL-1β protein for 48 h or preincubated with IL-1RA (1,000 ng/ml) for 1 h. Endothelial permeability was evaluated by the measurement of *trans-*endothelial electrical resistance. **(B–D)** C57BL/6 mice received tail vein injection of 300 μl PBS (control group, *n* = 8) or injection of 300 μl of PBS containing 0.2 μg of recombinant mouse IL-1β protein (IL-1β group *n* = 8) or were pretreated with intraperitoneal injection of 300 μl of PBS containing 2 μg of human IL-1RA at 90 min before the tail vein injection of 300 μl of PBS containing 0.2 μg of recombinant mouse IL-1β protein (IL-1RA + IL-1β group, *n* = 8). At 9 h postinjection with IL-1β, mice were euthanized, and the distribution of IL-1β in the heart, liver, spleen, lung, large intestine, and small intestine was analyzed by immunohistochemistry. Black arrows indicate the immunostaining of IL-1β **(B)**. Histopathologic analysis of the heart, liver, spleen, lung, large intestine, and small intestine. Black arrows indicate infiltrating inflammatory cells **(C)**. At 9 h after the administration of IL-1β, mice were intravenously injected with Evans blue. The dye was allowed to circulate for 2 h before mice were euthanized, and liver, spleen, lung, large intestine, and small intestine were collected. The concentration of Evans blue was measured at OD_610_ **(D)**. Data represent two independent experiments. Values are mean ± SEM; ^∗^, ^∗∗^, ^∗∗∗^ indicate *P-*values less than 0.05, 0.01, and 0.001, respectively.

**FIGURE 7:**
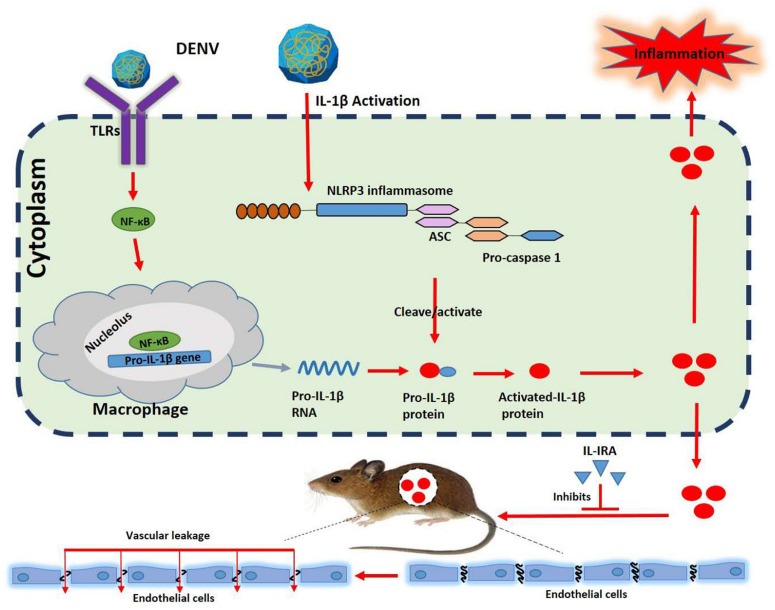
A distinct mechanism by which dengue virus (DENV) activates interleukin (IL)-1β to induce tissue injury and vascular leakage. Upon DENV infection, the DENV promotes ASC oligomerization and inflammasome assembly. The inflammasome facilitates the synthesis and secretion of IL-1β, inflammation, and vascular leakage. The present study highlights the major contribution of IL-1β to DENV-associated pathology.

Finally, the effect of IL-1β on endothelial cell permeability was examined *in vitro* to demonstrate whether IL-1β can directly induce vascular leakage. For this purpose, HUVECs were treated with recombinant human IL-1β protein or preincubated with human IL-1RA (Descamps et al., 2012), and the endothelial permeability was determined by the measurement of TEER. The magnitude of TEER level was reduced by the treatment of cells with IL-1β, but this decrease was reversed in the presence of IL-1RA (Figure 6A). Thus, IL-1β induces endothelial hyperpermeability in HUVECs. However, it should be noted that these *in vitro* experiments utilized concentrations of IL-1β higher than those measured in DENV2-infected mice. Recombinant IL-1β was intravenously injected into the caudal region of C57BL/6 mice or preincubated with mouse IL-1RA through intraperitoneal injection (Itani et al., 2016). The injection of IL-1β or IL-1RA + IL-1β resulted in a significant increase in circulating IL-1β mRNA and IL-1β protein in comparison with PBS-treated mice (Supplementary Figures 5A,B), indicating that IL-1β was successfully delivered. IL-1β-injected mice exhibited higher tissue levels of IL-1β, barely detectable in the tissues of IL-1β + IL-1RA-treated mice (Figure 6B). Similarly, infiltration by inflammatory cells and tissue injury were present in the tissues of IL-1β-treated mice but not in IL-1RA + IL-1β-treated mice (Figure 6C). The vascular leakage assay demonstrated that the intensity of Evans blue was significantly higher in the tissues of IL-1β-treated mice but relatively unaltered in the tissues of IL-1RA + IL-1β-treated animals (Figure 6D), supporting the notion that IL-1β induces vascular leakage in mice. Taken together, these results demonstrate that IL-1β plays an important role in the induction of inflammatory response and vascular leakage (Figure 7).

## Discussion

Immune dysfunction during DENV infection may cause life-threatening hypovolemic shock resulting from the leakage of vascular fluids, and the ensuing cytokine storm is thought to contribute to the pathogenesis of DENV (Culshaw et al., 2017). While this process has been partially characterized, the specific mechanisms governing cytokine regulation upon DENV infection remain largely unknown. The current work provides experimental evidence of the activation of IL-1β synthesis and secretion during vascular leakage in DENV-associated infection and identifies IL-1RA as a potential agent for the prevention and treatment of this infectious disease. Although it was previously shown that DENV infection induces the production of IL-1β (Hottz et al., 2013; Wu et al., 2013), the present findings strengthen this conclusion by using samples from DENV patients and a mouse model of DENV infection, primary cells isolated from infected mice, PBMCs isolated from healthy individuals, and *in vitro* cultures of human macrophages infected by two different strains of DENV. Moreover, the obtained results show that DENV increases the serum concentration of IL-1β in both patients and mice, strongly suggesting that DENV activates IL-1β *in vivo*.

IL-1β is an essential proinflammatory cytokine acting as a fever-inducing pyrogen in the host (Kugelberg, 2016; Leake, 2019). *In vivo*, the NLRP3 inflammasome is required for IL-1β processing, with caspase -1 and ASC oligomerization mediating IL-1β maturation and secretion. As a crucial element of the innate immune system, the NLRP3 inflammasome is not only an important component in host defense against pathogens (Allen et al., 2009; Wang et al., 2018; Negash et al., 2019) but is also involved in the progression of several inflammatory human diseases, such as gout and type 2 diabetes (So and Martinon, 2017; Wu et al., 2018). Additionally, DENV infection triggers the assembly of the NLRP3 inflammasome in platelets, further contributing to increased vascular permeability by synthesis and release of IL-1β (Hottz et al., 2013). However, the mechanism underlying the relationship between enhanced inflammatory response and loss of vascular barrier integrity has not been addressed *in vivo*. The current work utilized the *IFNAR*^–/–^ C57BL/6 mouse model of DENV infection, and documented high serum levels of inflammatory factors IL-1β and TNF-α were abundantly expressed in sera. IL-1β was also highly expressed in different organs, including heart, liver, spleen, lung, large intestine, and small intestine. These alterations in the expression and distribution of cytokines were accompanied by a significant change in vascular permeability. Importantly, IL-1RA alleviated the damage produced by inflammatory factors, demonstrating that IL-1β may have an important role in the pathogenesis of the dengue disease in a TNF-α-independent manner. The obtained results provided also evidence of the induction of vascular leakage in human HUVEC and C57BL/6 mice by recombinant IL-1β. Conversely, competitive binding of IL-1β receptor by IL-1RA ameliorated IL-1β-induced damages.

In conclusion, during DENV infection, the virus promotes the oligomerization of ASC and the assembly of inflammasome, facilitating the synthesis and secretion of IL-1β, and, thereby, activating inflammation, which may lead to vascular leakage. Thus, the presented investigation identified the contribution of IL-1β to DENV-associated pathology and established a possible direct link between IL-1β and vascular leakage. These findings suggest that IL-1β is an essential part of the complex immunopathological nature of severe dengue, and targeting this cytokine may be used in the prevention and treatment of DENV-associated diseases.

## Data Availability Statement

All datasets generated for this study are included in the article/Supplementary Material.

## Ethics Statement

The studies involving human participants were reviewed and approved by the Institutional Review Board of the College of Life Sciences, Wuhan University. The patients/participants provided their written informed consent to participate in this study. The animal study was reviewed and approved by the Institutional Review Board of the College of Life Sciences, Wuhan University.

## Author Contributions

PP, QZ, WL, WW, QT, KW, YiL, GL, and JW contributed to the design of the experiments. PP, QZ, WL, WW, ZY, ZL, WIZ, MS, PW, FX, and MS contributed to the conduction of the experiments. PP, QZ, WL, WW, ZY, ZL, WIZ, MS, PW, FX, MS, WNZ, and QT contributed to the reagents. PP, QZ, WL, WW, ZY, ZL, WIZ, MS, PW, FX, MS, and WNZ, and QT contributed to the analyses of the data. PP, QZ, WL, YiL, GL, and JW contributed to the writing of the manuscript. PP and JW contributed to the editing of the manuscript.

## Conflict of Interest

The authors declare that the research was conducted in the absence of any commercial or financial relationships that could be construed as a potential conflict of interest.
