# Supplementary material for: Dengue Virus Infection Activates Interleukin-1β to Induce Tissue Injury and Vascular Leakage
Source: Front Microbiol. 2019 Nov 22;10:2637. doi: 10.3389/fmicb.2019.02637 (PMC6883832; doi:10.3389/fmicb.2019.02637)
Supplement: Supplementary file 1 [file Data_Sheet_1.pdf]

Dengue Virus Infection Activates Interleukin-1β to Induce Tissue Injury and Vascular Leakage

Supplementary Material

Supplementary Methods

RNA extract and qRT-PCR.

Trizol reagent (Invitrogen, Carlsbad, CA) was used for total cellular RNA extracted according to the manufacturer's instructions. Then the RNA (1 μg) were reverse transcribed to cDNA with 0.5 μl oligo (dT) and 0.5 μl Random primer at 37°C for 60 min and 72°C for 10 min. The resulting cDNA was used as templates for real-time PCR analysis. Real-time PCR was performed in a LightCycler 480 thermal cycler (Roche) by the following procedure: heat activate polymerase at 95 for 5 min, 45 cycles of 95°C for 15s, 58°C for 15s and 72°C for the 30s, the fluorescence was collected and analyzed at the 72°C step. A final melting curve step from 50°C to 95°C was used to test the specificity of the primer. The primers used in real-time PCR detection were listed in Supplementary Table 2.

Enzyme-linked immunosorbent assay (ELISA).

The concentration of culture supernatants and serum of IL-1β were measured by IL-1β ELISA Kit (BD Biosciences, CA for Human and R & D systems (Minneapolis, MN for Mice) and the concentration of culture supernatants of LPS was measured by LPS ELISA Kit (EXPANDBIO) according to manufacturer's instructions.

Western-blot.

PMA-differentiated THP-1 cells were washed twice with PBS and dissolved in THP-1 lysis buffer (50 mM Tris-HCl, 150 mM NaCl, 0.1% Nonidetp40, 5 mM EDTA, and 10% glycerol, pH7.4). Protein concentration was measured by the Bradford assay (Bio-Rad, Richmond, CA). Cultured cell lysates (50 μg) were electrophoresed in an 8–12% SDS polyacrylamide gels and transferred to nitrocellulose membranes (Amersham, Piscataway, NJ). Nonspecific bands of NC membranes were blocked by using 5% skim milk for 2 h. Then membranes were washed three times with phosphate buffered saline with 0.1% Tween (PBST) and incubated with

the antibody. Protein bands were visualized using a Luminescent Image Analyzer (Fujifilm LAS-4000).

Immunofluorescence.

PMA-differentiated THP-1 cells were grown on sterile cover slips were infected with DENV2(NGC) (MOI=5) for 48 h. Cells were fixed with 4% paraformaldehyde for 15 min and then washed three times with wash buffer (ice-cold PBS containing 0.1% BSA), permeabilized with PBS containing 0.2% TritonX-100 for 5 min and washed three times with wash buffer, after blocking with 5% BSA for 30 min, cells were incubated overnight with anti-ASC antibody and anti-DENV-Prm antibody (1:200 in wash buffer) followed by staining with FITC-conjugated donkey anti-mouse IgG and Daylight 649-conjugated donkey anti-rabbit IgG secondary antibody (Abbkine) (1:100 in wash buffer) for 1 h. Nuclei were stained with DAPI for 5 min, and then the cells were washed three times with wash buffer. Finally, the cells were viewed using a confocal fluorescence microscope (Fluo View FV1000; Olympus, Tokyo, Japan).

ASC oligomerization analysis.

The PMA-differentiated THP-1 cells were lysed by lysis buffer (50 mM Tris-HCl, 150 mM NaCl, 1% Nonidetp40, 5 mM EDTA, and 10% glycerol, pH7.4). The lysates were gently shaken at 4°C for 30 min and centrifuged at 6000 rpm at 4°C for 15 min. The pellets were washed three times with PBS and re-suspended in 500 μl PBS. 2 mM DSS (Sigma) was added to the re-suspended pellets, which were cross-linked at 37°C for 30 min. The samples were then centrifuged at 6000 rpm for 10 min, and the cross-linked pellets were re-suspended in 50 μl 2 × SDS loading buffer, boiled for 10 min and analyzed by Western blotting.

Supplementary Figures and Legends

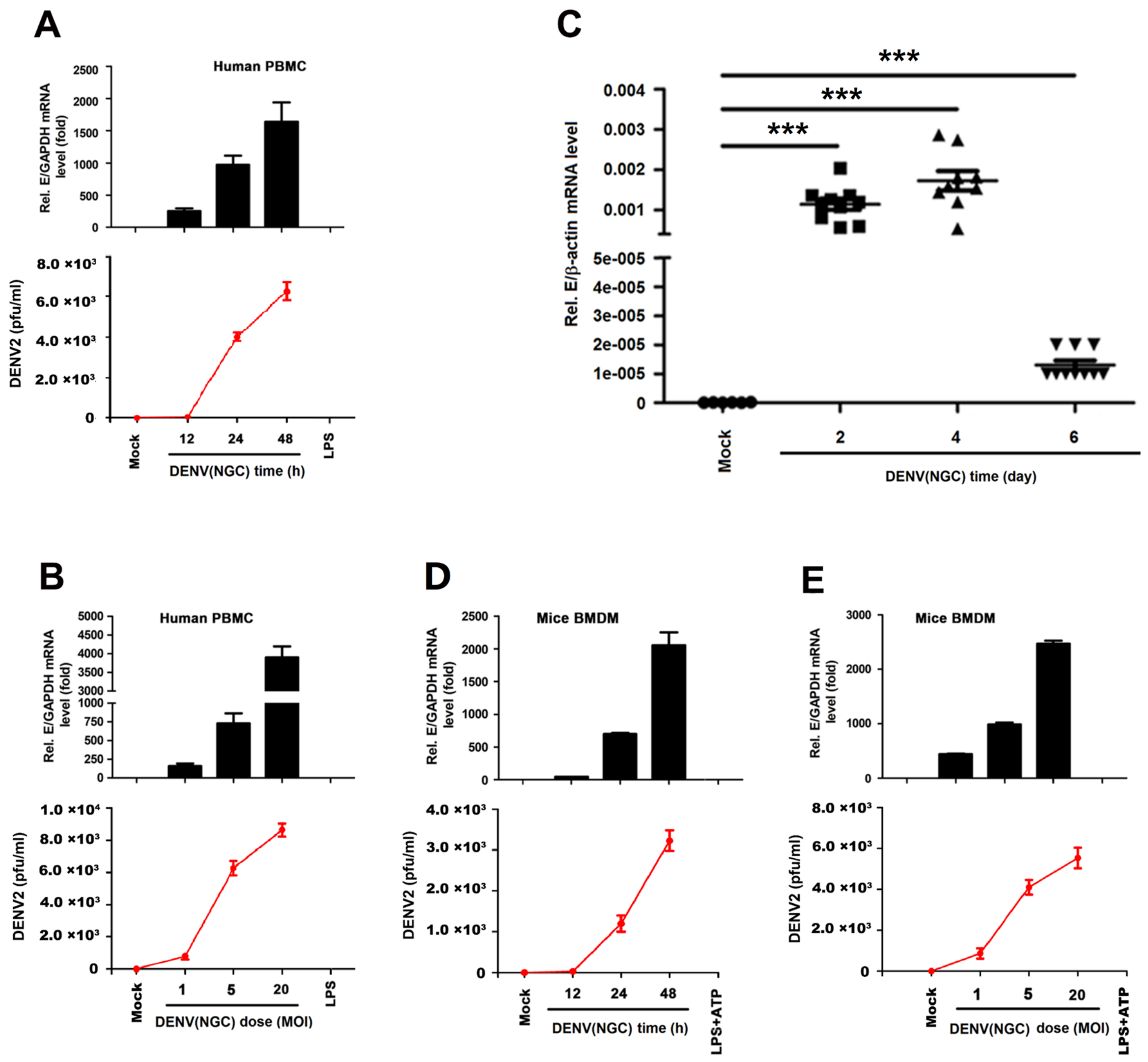

Supplementary Figure 1. DENV infection induced IL-1β activation in patients and mice. (**A**, **B**) Human PBMCs infected with DENV2(NGC) at MOI=5 for different times (12, 24, and 48 h) (A) or at different doses (MOI=1, 5, and 20) for 24 h (B). DENV *E* mRNA were extracted from cell and quantified by real-time PCR (RT-PCR) (top). DENV2 infectious virus were analyzed from supernatants by plaque assay (bottom). (**C**) Total RNAs were extracted from blood samples of mock-infected *IFNAR*$^{-/-}$ mice (n=6) and DENV2(NGC)-infected *IFNAR*$^{-/-}$ mice (n=8) at 2, 4, and 6 days post-infection. The DENV *E* gene mRNA was quantified by RT-PCR. (**D**, **E**) GM-CSF-differentiated BMDMs were infected with DENV2(NGC) at MOI=5 for different times (12, 24, and 48 h)

(D) or at different doses (MOI=1, 5, and 20) for 24 h (E). DENV *E* gene mRNA were extracted from cell and quantified by RT-PCR (top). DENV2 infectious virus were analyzed from supernatants by plaque assay (bottom). The number of replicates is three (A, B, D, and E). The number of replicates is two (C). Values are mean ± SEM, P ≤0.05 (*), P ≤0.01 (**), P ≤0.001 (***).

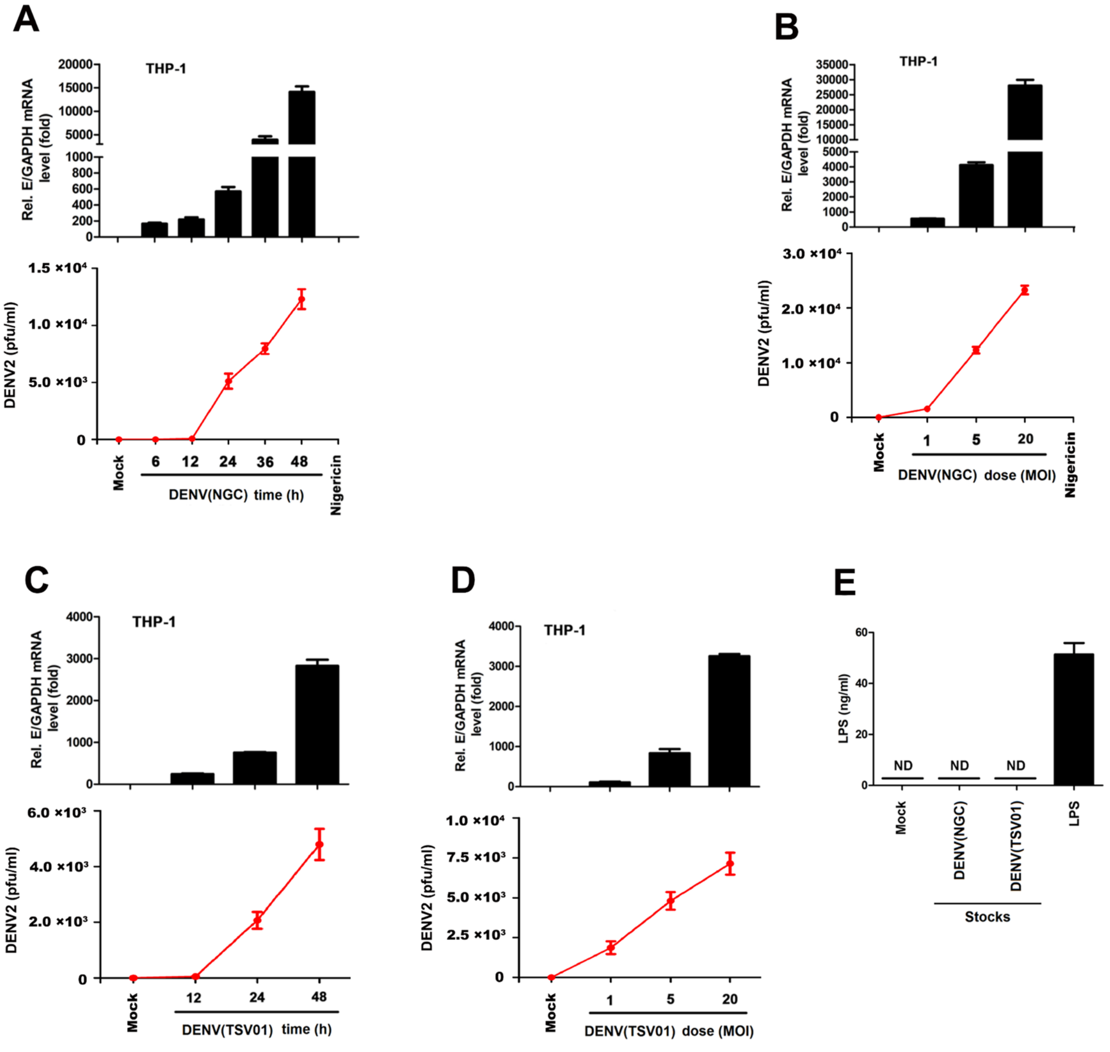

Supplementary Figure 2. Different strains of DENV activate IL-1β in THP-1 macrophages. (**A**, **B**) PMA-differentiated THP-1 macrophages were infected with DENV2(NGC) at MOI=5 for different times (6, 12, 24, 36, and 48 h) (A) or at different doses (MOI=1, 5, and 20) for 24 h (B). DENV2(NGC) *E* mRNA were extracted from cell and quantified by RT-PCR (top). DENV2(NGC) infectious virus were analyzed from supernatants by plaque assay (bottom). (**C**, **D**) PMA-differentiated THP-1 macrophages were infected with DENV2(TSV01) at MOI=5 for different times (12, 24, and 48 h) (A) or at different doses (MOI=1, 5, and 20) for 24 h (B). DENV2(TSV01) *E* mRNA were extracted from cell and quantified by RT-PCR (top). DENV2(TSV01) infectious virus were analyzed from supernatants by plaque assay (bottom). (**E**) DENV2(NGC) or DENV2(TSV01) infected supernatant of C6/36 cells was collected and LPS in the supernatant was measured by ELISA, mock-infected supernatant of C6/36 cells were used as negative

controls, commercialized LPS was used as a positive control. Mock means no DENV2 infected supernatant of C6/36 cells. The number of replicates is three. Values are mean ± SEM, $P \leq 0.05$ (*), $P \leq 0.01$ (**), $P \leq 0.001$ (***).

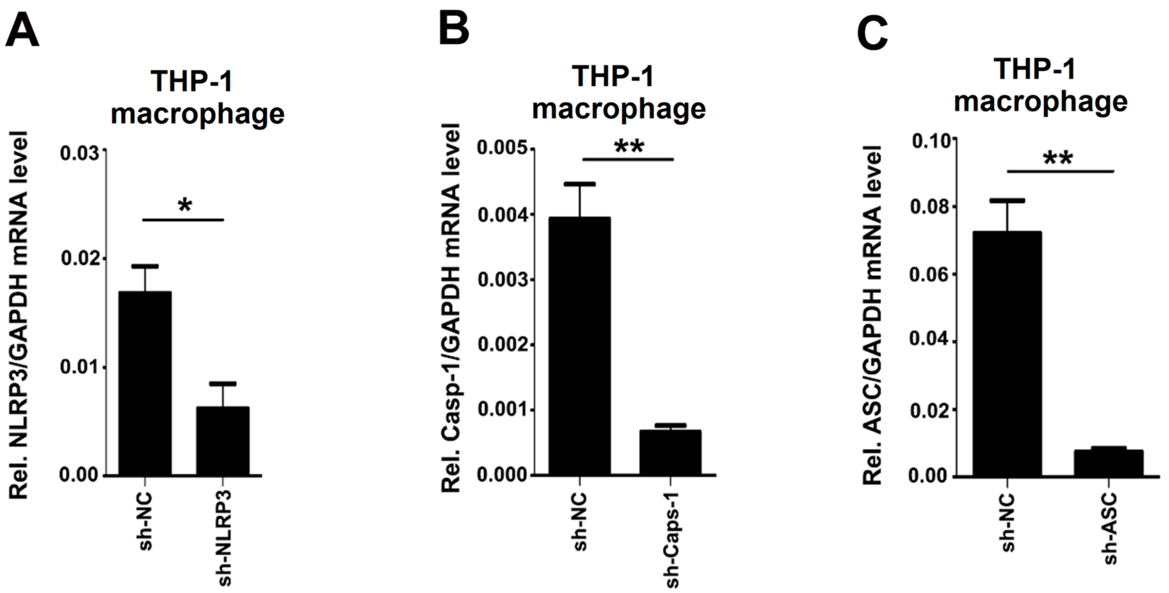

Supplementary Figure 3. DENV induces IL-1β release via activating the NLRP3 inflammasome. (**A–C**) The mRNA levels of *NLRP3* (A), *Caspase-1*(B), and *ASC* (C) were measured by RT-PCR in PMA-differentiated THP-1 macrophages stably expressing sh-RNAs, sh-*NLRP3*, sh-*ASC*, sh-*Casp-1*, or sh-NC.

Figure S4

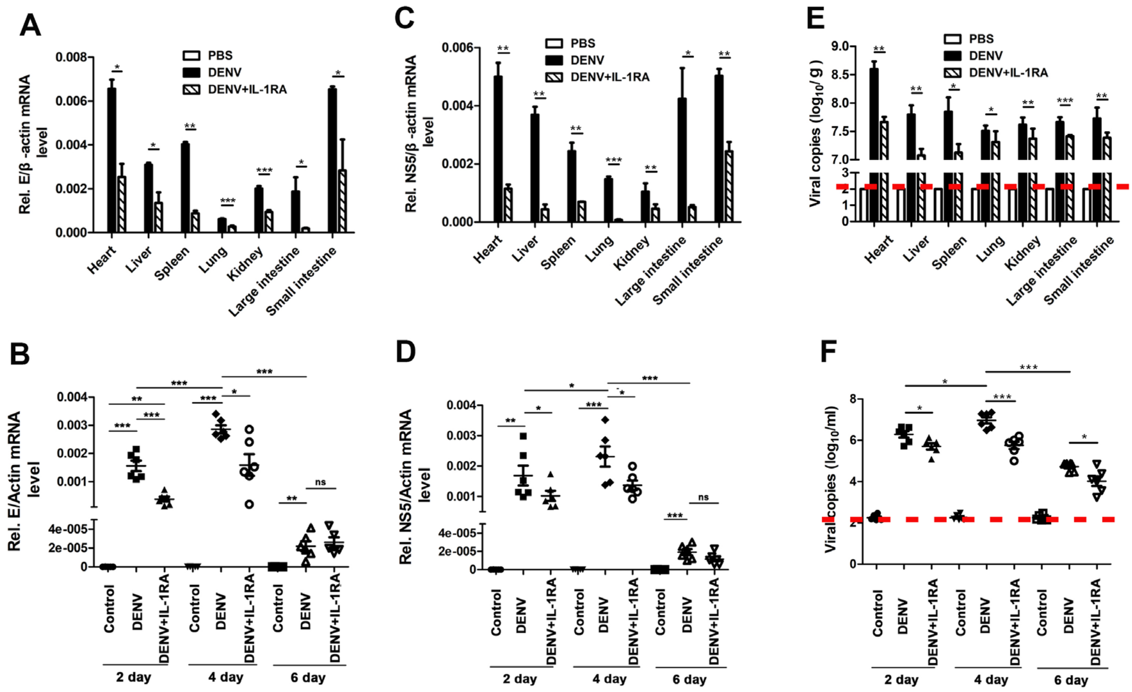

Supplementary Figure 4. DENV promotes IL-1β release in IFNAR$^{-/-}$ C57BL/6 mice. (**A–F**) *IFNAR*$^{-/-}$ C57BL/6 mice were intravenously injected with 300 μl DENV2 at a dose of 1×10^6 PFU/mouse (n=6), pre-treated with 300 μl PBS containing 2 μg mice IL-1RA by intraperitoneal injection for 90 min and then infected with DENV2 (1×10^6 PFU/mouse), repeat treated with IL-1RA (2 μg /mice) on the fourth day after DENV2 infection (n = 6) or 300 μl PBS as a control group (n=6). After 7 days post-infection, mice were euthanasia, and the tissues were collected. (**A**, **C**, and **E**) Total RNAs were extracted from tissues, including heart, liver, spleen, lung, kidney, large intestine, and small intestine at 6 days post-infection. DENV *E* gene mRNA (A), *NS5* gene mRNA (B), and viral copies (E) were quantified by RT-PCR. (**B**, **D**, and **F**) Total RNAs were extracted from blood samples at 2, 4, and 6 days post-infection. DENV *E* gene mRNA (B), *NS5* gene mRNA (D), and viral copies (F) were quantified by RT-PCR. Dates were representative of two independent experiments. Values are mean ± SEM, P ≤0.05 (*), P ≤0.01 (**), P ≤0.001 (***).

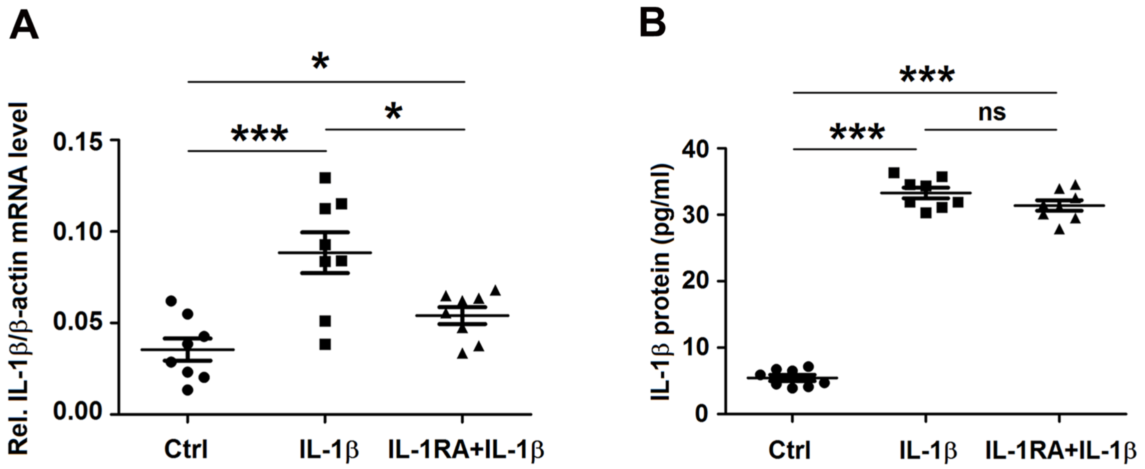

Supplementary Figure 5. Recombinant IL-1β induces directly vascular leakage in mice. (**A**, **B**) C57BL/6 mice were tail vein injected with 300 µl PBS as Control group (n = 8) or 300 µl PBS containing 0.2 µg recombinant mouse IL-1β protein as IL-1β group (n = 8) or pre-treated with 300 µl PBS containing 2 µg human IL-1RA by intraperitoneal injection for 90 min and then treated with 300 µl PBS containing 0.2 µg recombinant mouse IL-1β protein by tail vein injection as IL-1RA+IL-1β group (n = 8). At 9 h post-injection with IL-1β, sera were collected from the orbit. IL-1β in the sera was measured by ELISA (A). The total RNA was extracted from the blood cells of mice. The RNA level of IL-1β was quantified by qRT-PCR (B). Points represent the IL-1β value of each serum samples. Dates were representative of two independent experiments. Values are mean ± SEM. NS, not significant. $P \leq 0.05$ (*), $P \leq 0.01$ (**), $P \leq 0.001$ (***)

Supplementary Tables and Legends

Supplementary Table 1. Characteristics of DENV-infected patients.

**Characteristic**	**Control**	**Dengue**
Age, years	35 (20-50)	33 (21-62)
Gender, male (%)	10 (50%)	8 (53.3%)
Platelet count (10^9/L)	—	124 (64-175)
Hematocrit (%)	—	43.8 (34.7-51.3)
Hemoglobin (g/L)	—	145 (109-168)
Lymphocytes counts (10^9/L)	—	0.9 (0.27-1.63)
Monocyte (%)	—	14.7 (6.9-19.9)
AST (U/L)	—	33 (28-99)
ALT (U/L)	—	39 (27-67)
ALP (U/L)	—	71 (44-106)
The day of fever	—	4 (3-7)
Primary/Secondary status	—	Primary
PCR detection	Negative	Positive
IgG/IgM detection	Negative	Positive

Note: Date are expressed as median or number (%) AST, aspartate aminotransferase; ALT, alanine aminotransferase; ALP, alkaline phosphatase.

Supplementary Table 2. Primers used in this study.

Name	Forward	Reverse
Human IL-1β	5’-CACGATGCACCTGTACGATCA-3’	5’-GTTGCTCCATATCCTGTCCCT-3’
Human NLRP3	5’-AAGGGCCATGGACTATTTCC-3’	5’-GACTCCACCCGATGACAGTT-3’
Human Caspase-1	5’-TCCAATAATGCAAGTCAAGCC-3’	5’-GCTGTACCCCAGATTTTGTAGCA-3’
Human ASC	5’-AACCCAAGCAAGATGCGGAAG-3’	5’-TTAGGGCCTGGAGGAGCAAG-3’
Human GAPDH	5’-AAGGCTGTGGGCAAGG-3’	5’-TGGAGGAGTGGGTGTCG-3’
DENV2-E	5’-CATTCCAAGTGAGAATCTCTTTGTCA-3’	5’-CAGATCTCTGATGAATAACCAACG-3’
DENV2-NS5	5’-AGAGCCCGAATTTCCCAAGG-3’	5’-TCCACATTTGGGCGTAGGAC-3’
Mouse TNF-α	5’-CCAAATGGCCTCCCTCTCAT-3’	5’-CAATAGTGATGACCTGGCCGT-3’
Mouse β-actin	5’-AGAGGGAAATCGTGCGTGAC-3’	5’-CAATAGTGATGACCTGGCCGT-3’
Mouse IL-1β	5’-TGCCACCTTTTGACAGTGATG-3’	5’-TGTGCTGCTGCGAGATTTGA-3’

Supplementary Table 3. Details of each dengue patients.

Sample code	DV-1	DV-2	DV-3	DV-4	DV-5	DV-6	DV-7	DV-8	DV-9	DV-10	DV-11	DV-12	DV-13	DV-14	DV-15
Sex	M	M	F	M	M	M	F	F	F	F	M	M	F	F	F
Age	62	56	33	32	43	24	31	49	34	40	24	27	24	42	21
WBC (10^9/L)	3.44	2.9	2.31	2.04	3.66	1.96	1.81	2.87	4.76	1.17	3.15	1.7	3.3	6.45	1.43
NEUT%	77.2	26.6	53.3	51.5	43.5	49.4	51.9	45.7	71.8	61.6	40.3	41.3	55.4	62	55.9
LYM%	7.9	56.1	39	40.2	39.3	28.1	30.4	34.5	20.1	25.6	46.8	38.2	29.1	21.4	28
MONO%	14.9	13.8	6.9	7.8	16.4	19.9	17.7	17.8	7.7	12.8	11.6	13.5	15.1	14.7	14.7
EOSTN%	0	2.7	0.4	0.5	0.3	2.6	0	0.3	0	0	0.5	2.9	0	1.9	0.7
BASO%	0	0.8	0.4	0	0.5	0	0	1.7	0.4	0	0.8	4.1	0	0	0.7
NEUT (10^9/L)	2.66	0.77	1.23	1.05	1.59	0.97	0.94	1.31	3.43	0.72	1.27	0.7	1.83	4	0.8
LYM (10^9/L)	0.27	1.63	0.9	0.82	1.44	0.55	0.55	0.99	0.96	0.3	1.48	0.65	0.96	1.38	0.4
MONO (10^9/L)	0.51	0.41	0.16	0.16	0.6	0.39	0.32	0.51	0.37	0.15	0.37	0.23	0.51	0.95	0.21
EOSIN (10^9/L)	0	0.07	0.01	0.01	0.01	0.05	0	0.01	0	0	0.01	0.05	0	0.12	0.01
BASO (10^9/L)	0	0.02	0.01	0	0.02	0	0	0.05	0.02	0	0.02	0.07	0	0	0.01
RBC (10^9/L)	4.3	5.4	5.94	4.12	5.08	5.18	4.56	4.62	5.63	4.83	4.42	4.89	4.67	4.42	5.38
Hb (g/L)	135	155	109	129	137	150	145	146	168	153	133	147	145	144	155
HCT%	39	45.2	34.7	39.4	43.8	46.1	41.7	42.6	51.3	45.1	40.3	44.2	43.6	43.8	45.9
MCV (FL)	90.6	83.7	58.4	95.6	84.3	89	89.5	92.2	91.1	93.4	91.2	90.4	93.4	99.3	85.3
MCH (pg)	31.4	28.6	18.4	31.3	27	29	31.1	31.6	29.8	31.7	30	30.1	31	32.8	28.8
MCHC (g/L)	346	342	314	327	320	325	348	343	327	339	329	333	333	328	338
RDW	12.8	13.6	18.9	12.4	12.8	12.2	11.4	11.6	12.7	11.8	11.7	12.5	12.6	11.2	12.2
PLT (10^9/L)	175	141	167	87	152	55	150	106	91	64	113	69	130	142	124
MPV (FL)	9.5	10.6		11.3	9.9	11.9	9.6	9.5	12.5	10.7	10.9	10.8	10	9.9	10
PCT%	0.17	0.15		0.1	0.15	0.07	0.14	0.1	0.11	0.07	0.12	0.07	0.13	0.14	0.12

**PDW**	15.9	16.1		12.2	10.6	14.3	10.5	10.4	15.9	14.6	116.4	11.8	10.3	10.9	11
**ALT (U/L)**	33	33	48	27	39	39	18	67	41						
**AST (U/L)**	32	33	44	28	25	76	28	99	45						
**ALP (U/L)**	80	106	71	44	73	58	46	75	70						
**GCT (U/L)**	67	16	20	16	32	27	28	42	12						
**TP (g/L)**	81.9	72.5	77.2	78.4	86.2	75.4	75.1	81.1	71.6						
**ALB (g/L)**	49.9	42.7	44.8	47	48.9	44.7	43.5	45.8	44.1						
**TBIL (μmol/L)**	9.4	8.7	10.5	4.1	15.6	4.5	9	23.7	8.6						
**Bc (μmol/L)**	0	0	0	0	0	0	0	0	0						
**Bu (μmol/L)**	6.6	3	7	1.2	6.9	3.7	6	16.9	3.2						
**IL-1β (pg/ml)**	20.90423	22.47771	22.32036	21.21892	23.89384	25.62467	26.09671	28.45693	30.03041	24.20854	26.7261	25.62467	24.05119	22.32036	22.63506
**The day of fever**	5	3	7	3	4	5	4	4	4	6	5	3	6	3	4
**Primary/ Secondary**	Primary	Primary	Primary	Primary	Primary	Primary	Primary	Primary	Primary	Primary	Primary	Primary	Primary	Primary	Primary
